# Round the Hospitals

**Published:** 1920-01-10

**Authors:** 


### ROUND THE HOSPITALS.

Now that nurse registration by Act of Parliament has become a factor in England and Wales, Scotland and Ireland, it is of the first importance that every fully-trained nurse should consider and decide for herself which of several steps is best calculated to promote her own interests as a working nurse. She will doubtless realise that during the earlier years of nurse registration the State registers will be flooded by nurses who cannot show the hall-mark of a first-class training. These nurses may, however, prove to be eligible for public appointments from which they have been hitherto excluded. These, and other considerations, may lead the fully-trained nurse, who is also a woman of business, first to take steps to secure her own position with the medical profession and the public, to whom she will look for remunerative work. If she is already registered with the College of

**ROUND THE HOSPITALS—(*continued*).**

nursing her certificate will at once put her in a commanding position as a trained nurse of the highest type. It is possible, therefore, that fully-trained nurses throughout the country will make it their business at once to register with the College of Nursing, and so to take rank, as the highest type of the trained nurse, whose services the public and and profession can accept with absolute confidence. In Scotland the nurse who holds the highest and best certificates of training has been handicapped by the treatment extended to the L.G.B. nurses and the nurses who cannot show the hall-mark of a first-class training in the Nurses' Registration (Scotland) Bill. If they are on the Register of the College of Nursing their rank as a trained nurse with the public should be highest possible. For this reason every fully-trained Scotch nurse will probably lose no time in registering with the College if she has not already done so. It is very regrettable that the clauses in question in the Nurses' Registration (Scotland) Bill should have been so muddled. Trained nurses everywhere should clearly understand, at the outset, that the Registration Act is in no sense a concession to the nursing profession any more than the Medical Acts were, or are, to that of medicine. The more business-like the nurse the more deliberate is her attitude towards entering her name on the State Register likely to prove in practice.

The period of armistice after the war has been marked by many curious instances of a perverted sense in individuals. The nurses on the nursing staff of the greatest nurses' co-operation in the world are credited, as we go to press, with an attack of hesitation which may result in suicide. The members of this co-operation have invited the nurses on the nursing staff, after perusing a strikingly impartial statement of facts which they have circulated, to vote "yes" or "no" as to whether the nurses will uphold the present constitution of the Nurses' Co-operation (22 Langham Street, W. 1) or not. If the majority of the nurses on the nursing staff vote "yes," the Nurses' Co-operation will continue on its prosperous way, and should renew its strength, to the advantage and comfort of all its nurses. Should only a minority of the nursing staff vote "yes," or vote at all, the members advise the nurses that the only course open to them will be to wind up the co-operation and take the directions of the Courts as to the disposal of the offices, the Home, and the other assets. We are glad to hear that every intelligent nurse on the nursing staff has decided to vote "yes" with both hands.

The subject of this week's meeting of the London Centre of the College, held in the rooms of the Medical Society, 11 Chandos Street, W. 1, at 8 p.m. on January 8, is one too much neglected by nurses. It deals with "Public Speaking: The Voice, its Possibilities and Powers." It is not too much to say that the whole nursing profession is held back from the exercise of its legitimate influence by the reluctance which even now keeps back many of its leading members from allowing their voices to be heard. The dictum "Deeds, not words" has been followed too literally. The result is that any firebrand, any ignorant pretender, is allowed to hold the floor, and with glib rhetoric to outface women who have given their lives to the study of difficult problems, yet lack the courage to stand up and defend their convictions of the truth. No College Centre can be considered properly equipped in its work which does not offer its members regular opportunities for "saying a few words" with composure and precision which can be heard at a distance of some yards and listened to with pleasure. We trust Miss Bagley, head of the Polytechnic School of Speech-training, has a large audience.

Best wishes for the success of the impending "Bulletin" of the College of Nursing. It was certain that some form of regular printed communication from the College to its numerous members must sooner or later come into existence, for there are many reports and items of intelligence with which it is important all members should be officially acquainted. We trust it may be found possible to distribute the paper largely through the agency of local centres or affiliated institutions, and thus save some of the great labour and expense of directing and posting it to individuals. The first number is awaited with much interest.

The local centres of the College of Nursing are giving ever-fresh practical evidence of the latent talents for organisation possessed by nurses outside their professional work. The determination of the Bristol Centre to establish a Nurses' Hostel in the city is the outcome of long consultation over the immediate needs of nurses, and is likely to bear fruit before the year is over. A series of entertainments has been arranged with the twofold object of providing money for the new enterprise and of interesting the public in the College and its aims. The grand concert in the Prince's Theatre on January 14 at 2.30 is to be followed up by an invitation concert the following day, held at the Bristol Royal Infirmary at 6 p.m., when all members of the Centre and their friends will be welcomed. The policy of enlisting the zeal, first of members of the College, next of all nurses, and lastly of the public, in the cause of the College of Nursing is a very sound one, and the results are already very satisfactory.

It is of great value to the nursing world that some of the leading daily papers are admitting well-informed articles on the prospects open to women who enter the nursing profession. In particular we are struck with an ably written article in the *Daily Telegraph* of December 27 on the attractiveness to educated women of the career of Queen's Nurse. Considerable damage has been caused by indiscriminate railing in the Press against the hardships of the nurse's lot, and good service is rendered by publishing exact information. We agree heartily

**ROUND THE HOSPITALS**—(*continued*).

that, considering the value of the work to the public, its immediate advantages, and the opportunities of promotion it offers, it is remarkable that more women from the Voluntary-Aid Detachments have not taken it up. They would undoubtedly find in it a splendid way of continuing their patriotic service during peace.

The existence of nursing sisters disabled and pensioned owing to former wars is not very vividly before the public mind. The rate of pension was very different from that which now prevails, and it is satisfactory to learn that the War Office is not oblivious of the hardships thus entailed. Announcement is made that the pension being paid on April 1, 1919, under the warrant of December 1, 1914, or any former warrant to any member of the Nursing Services in respect of a disability due directly and wholly to service in a former war may be increased by the difference between such pension and the pension which she might receive according to the degree of her disablement as certified under a warrant of August 1, 1917, if she were subject to the provisions of this warrant. The new warrant has effect as from April 1, 1919. We cannot say that the style impresses us as an example of lucid composition, but the underlying intention is clearly good.

The upward tendency of salaries observable in this country has extended to the Dominions, where for various reasons, however, salaries have always ruled considerably higher than over here. We understand that the ward sisters at the Melbourne Hospital, whose salary has hitherto been £70-£80, are for the future to receive £80-£100, with board, residence, uniform, laundry, and allowance for annual holiday. This is quite good, and should suffice to attract and retain a high class of sisters most necessary in this great training centre.

The 218th London V.A.D. has received special marks of public gratitude on account of the great courage displayed by the members in the hospitals when Lewisham and the adjoining districts were being heavily bombed from the air. A fine group of the Lewisham V.A.D. nurses is given in the *Sphere*. The Mayoress of Lewisham presented them all with gold medals and paid a high tribute to the spirit of devotion to duty they displayed. It was a pretty ceremony, and one calculated to strengthen the desire to render voluntary service to the community. While tolerably indifferent to the plaints of the V.A.D.s. who nurse as a grievance the fact that they have been insufficiently thanked, we are struck with the diversity which has attended the break-up of the voluntary war organisations. In some Red Cross Divisions a cordial spirit of friendliness has prevailed and the members suspended their activities with real regret and a determination to do more. In others the authorities, to say the least, have been less happy in their farewells. It is quite clear by this time that no one is particularly flattered by the receipt of printed or lithographed letters of thanks. Something "to stick in the coat" pleases better.

A "Provincial Matron," writing in our last issue, strongly enforces the contention, often urged in these columns, that the registration of the trained members of the Nursing Services needs to be supplemented by the licensing of the untrained or partially trained women who do nursing work. The number of nursing sisters eligible for registration is probably less than half the number of those who do nursing work with but very slender qualifications. Many of these women have a strong claim to consideration, having been assisted out of public funds to acquire the smattering of nursing lore which they possess. Everyone admits that in the present state of the Nursing Service they are indispensable, for they undertake work which trained women decline. It would be no hardship to them were they compelled to take out an annual licence from the municipal authority at a charge of, say, 2s. 6d., authorising them to work in a defined area, and undertake specified duties for the sick. By this means the entire Nursing Service could be got under control. Without some such auxiliary system of licensing registration will expose us to the anomaly of keeping guard over the efficient and letting the rest do as they please.

The problem of feeding the staffs in large institutions has become greatly complicated by the shortage of labour, and complaints about food largely centre round bad waiting and consequent confusion, with the natural result that the food is served lukewarm. The canteen system has many advantages, and we note, in the December number of the *American Journal of Nursing*, that the Bellevue Training School for Nurses has introduced it under an improved system, with admirable results for the convenience of the 400 nurses in the school. The canteen is known as a cafeteria. It was installed in a large room adjoining the dining-room last May. The equipment includes mould tables for cold and hot food; heated cabinets; a steam table, soup and vegetable jars, coffee urns, etc. The counter is covered with a hood of galvanised iron fitted with panels of wired glass. There is a large refrigerator, with cold storage. A line of 200 nurses can be served in fifteen minutes, each nurse securing her own little tray, flat silver, and desired food, and returning with it to her table. Waitresses keep the tables cleared of soiled dishes. It is claimed that the cafeteria system eliminates waste, ensures prompt service, and is popular.

The demand for public health nurses in America since the war is unparalleled. The Red Cross Bureau, which undertook the resettlement of war nurses, has been receiving wholesale requests. North Carolina applied for 21 nurses; the Tuberculosis Association in Illinois wanted 50; the State Department in one of the western States petitioned for 150 public health nurses. A high educational

**ROUND THE HOSPITALS**—*(continued)*.

standard is demanded, many towns demanding that their nurses shall be college graduates. We could wish that the British municipalities were as eager to secure the best kind of nurse as the towns over the water.

We are requested to state that Miss Miller is holding Bible meetings through January in the Fyvie Hall, Polytechnic, Regent Street, W.1., at 7.45 P.M. Miss Miller is well known as an interesting lecturer, always ready to extend a special welcome to nurses.

The war has made a notable difference in the amount of accommodation available in convalescent homes. In an endeavour to procure accommodation for a nurse invalided after diphtheria, but, of course, now free from infection, we have had letter after letter announcing that the home to which we made application was closed. Some, like the Clara Baroness de Hirsch Home, on Hampstead Heath, have been absorbed as military hospitals, and have not yet resumed normal activities. Others, like the Merchant Taylors' charming home for ladies at Bognor, are closed altogether. It is certain that there must be a large extension of convalescent treatment when the Health Ministry gets into its stride. The need is for exact knowledge in each district of the available accommodation for all classes of patients, and particularly for more facilities to be offered for convalescence after infective complaints. Some central bureau in each county for sorting cases and procuring information as to vacancies would save much useless correspondence.

The controversy about the introduction of female nurses into the mental hospital in Whitchurch, Cardiff, is still proceeding. The Cardiff Trades and Labour Council have now taken it up, and have written to the Home Secretary and to the Corporation asking that female nurses may not be introduced, on the ground that the presence of women nurses in the male lunatic wards has an injurious effect upon the inmates, both from the point of view of discipline and morals. The rather sensational evidence adduced in support of the contention seems to us to lack confirmation. We confess ourselves uncertain whether the agitation against continuing the services of women nurses in the mental hospital, where they proved their worth during the war, is really due to regard for the patients' welfare. It would seem there is some fear lest women should invade a province hitherto occupied by men. If the Cardiff Corporation has a sufficiency of both male and female candidates competing for service in the mental hospital they are extremely fortunate. It might not be amiss to turn the thoughts of some of these applicants towards medical and surgical nursing.

An article on the "Lost Worker" by the medical correspondent of the *Times* brings into prominence the special difficulty which attaches to every form of female employment, that of inducing the worker to remain long enough to rank as trained and efficient. Statistics have been collected from numerous workshops and factories tending to show the restlessness and caprice which haunts the young women of the day in selecting a sphere of work. The records have been carefully classified and the causes of leaving prematurely are shown under three headings, viz.—(1) Ill-health; (2) Sufficient reason other than ill-health; (3) No reason, or an insufficient one. Out of a total number of about 11,000 women who left, approximately 6,700 gave no reason for doing so. The tendency to drift about on the part of female workers is plainly responsible for the reluctance shown to make a formal three-year engagement to enter hospitals as probationers. The notion of being tied for a long period is repugnant to workers whose powers of self-restraint have been weakened by the conditions of war work. The women who should now at twenty-one or twenty-two be applying to become probationers have been for six years exposed to unusual excitement and a bewildering choice of work. As many as 132 per 1,000 girls during the war period were found not to outstay the second month.

News comes very rarely from the typhus-stricken regions of middle Europe. Lately the curtain has been lifted for a moment and we learn that The Friends' Emergency and War Victims' Relief Committee have received news of the death of two of their relief workers in Poland, Mr. R. Reynolds Ball and Miss Powicke, both from typhus, within a few days of each other. They were working in the infected district near Lemberg when they were attacked by the fever. Two other members of the unit who were also attacked are making a good recovery in hospital. There are sixteen other workers on duty from the same corps combating the disease. Miss Powicke was formerly on the staff of the Manchester Girls' High School. She volunteered at the beginning of the war, spent a year learning nursing and motor driving and did good service in France before she went to Poland last July. She was only thirty-one years of age. A terrible account of the typhus epidemic in Siberia has also come through.

Is it not time that something was done to stop the wastage in matrons through overwork? At the present moment two matrons of exceptional ability have been compelled to retire prematurely from work, the one at Kingston on account of injury to health caused "by the arduous nature of her duties," the other at Hampstead by reason of "severe nervous exhaustion." It is not creditable to the Guardians in either locality that both these ladies should have broken down in Poor-Law institutions. A timely rest, an extra sister as assistant, a little ordinary consideration, and these valuable workers, both of whom are deeply regretted by their nurses, could have continued at their labours in the full enjoyment of active work. We hope that under the Ministry of Health the crime of wearing out the matron will suffice to place a black mark against the managers of an institution. Lord Fisher's classic remedy would seem to fit the case.

